# Volumetric segmentation in the context of posterior fossa-related pathologies: a systematic review

**DOI:** 10.1007/s10143-024-02366-4

**Published:** 2024-04-19

**Authors:** Andrew J. Kobets, Seyed Ahmad Naseri Alavi, Samuel Jack Ahmad, Ashley Castillo, Dejauwne Young, Aurelia Minuti, David J. Altschul, Michael Zhu, Rick Abbott

**Affiliations:** 1 Department of Neurological Surgery, Montefiore Medical Center and the Albert Einstein College of Medicine, Bronx, NY 10467 USA; 2 Albert Einstein College of Medicine, New York City, USA

**Keywords:** Posterior fossa, Posterior fossa volume, Chiari malformation, Segmentation

## Abstract

**Background:**

Segmentation tools continue to advance, evolving from manual contouring to deep learning. Researchers have utilized segmentation to study a myriad of posterior fossa-related conditions, such as Chiari malformation, trigeminal neuralgia, post-operative pediatric cerebellar mutism syndrome, and Crouzon syndrome. Herein, we present a summary of the current literature on segmentation of the posterior fossa. The review highlights the various segmentation techniques, and their respective strengths and weaknesses, employed along with objectives and outcomes of the various studies reported in the literature.

**Methods:**

A literature search was conducted in PubMed, Embase, Cochrane, and Web of Science up to November 2023 for articles on segmentation techniques of posterior fossa. The two senior authors searched through databases based on the keywords of the article separately and then enrolled joint articles that met the inclusion and exclusion criteria.

**Results:**

The initial search identified 2205 articles. After applying inclusion and exclusion criteria, 77 articles were selected for full-text review after screening of titles/abstracts. 52 articles were ultimately included in the review. Segmentation techniques included manual, semi-automated, and fully automated (atlas-based, convolutional neural networks). The most common pathology investigated was Chiari malformation.

**Conclusions:**

Various forms of segmentation techniques have been used to assess posterior fossa volumes/pathologies and each has its advantages and disadvantages. We discuss these nuances and summarize the current state of literature in the context of posterior fossa-associated pathologies.

## Introduction

Segmentation of medical imaging enables researchers and clinicians to assess anatomical structures in both qualitative and quantitative manners. By separating specific target tissues from the surrounding anatomy, one may evaluate the etiology or prognosis of a disease more accurately thereby facilitating treatment and improving patient outcomes [1]. The posterior fossa or posterior cranial fossa of the brain remains a relatively fertile target for researchers and the segmentation method has already helped elucidate the mechanics of conditions such as Chiari malformations. Chiari malformation type I (CM-I), the most common form of Chiari malformation, involves cerebellar tonsillar herniation through the foramen magnum, with associated impairment of cerebrospinal fluid (CSF) flow [2, 3]. Syringomyelia is found in 30 to 70 percent of patients with (CM-I) and they generally present as young adults, suffering from cervical pain and headaches. However, up to 30 percent of patients may be asymptomatic [2–4]. Patients with CM-I have also been found to possess small posterior fossa. The crowding theory posits that the herniation of the hindbrain through the foramen magnum results from underdevelopment of the posterior fossa and occipital bone [2, 3, 5]. Chiari malformation type 2 involves the caudal herniation of the medulla, inferior vermis, and fourth ventricle. Patients commonly present in infancy and suffer from hydrocephalus, apnea, and feeding difficulties. Besides, concomitant myelomeningocele is commonly associated with Chiari malformation type 2 [6, 7]. Additionally, Other rarer forms of Chiari malformation include types 3 and 4. Chiari type 3 involves the existence of an occipital encephalocele and a poor prognosis. Patients with Chiari type 4 have cerebellar hypoplasia without any cerebellar herniation [8].

**Fig. 1:**
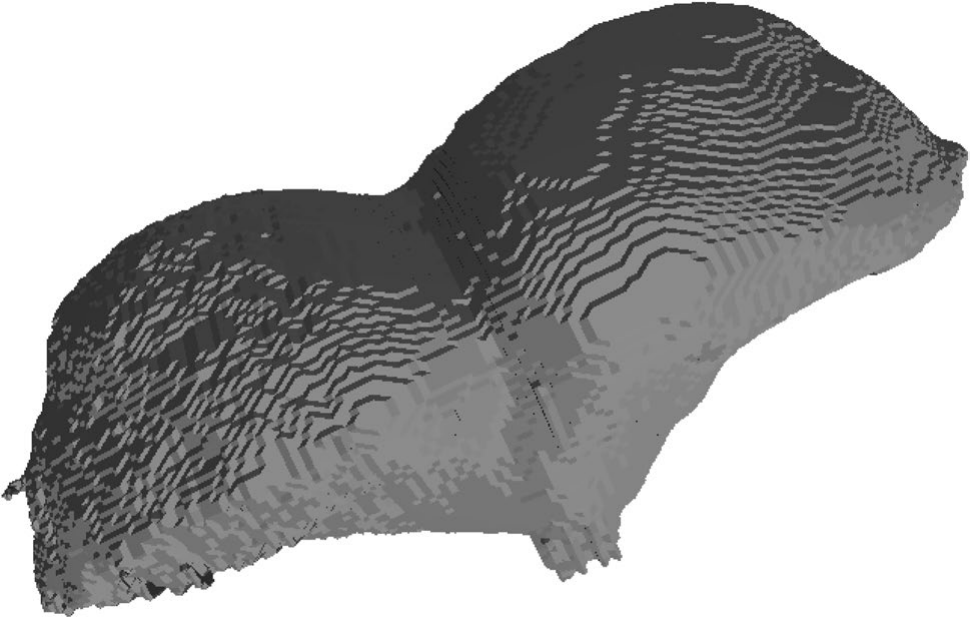
Posterior Fossa Volume stl file

Measurements of posterior fossa volume (PFV) (Fig. 1) were initially carried out by geometric calculations using 2D parameters obtained from skull radiographs or midsagittal MR images [9]. However, segmentation of the posterior fossa to conduct volumetric and morphological analysis of brain tissues in fetuses, though challenging due to imaging-related artifacts, has still been complicated [10].

**Fig. 2:**
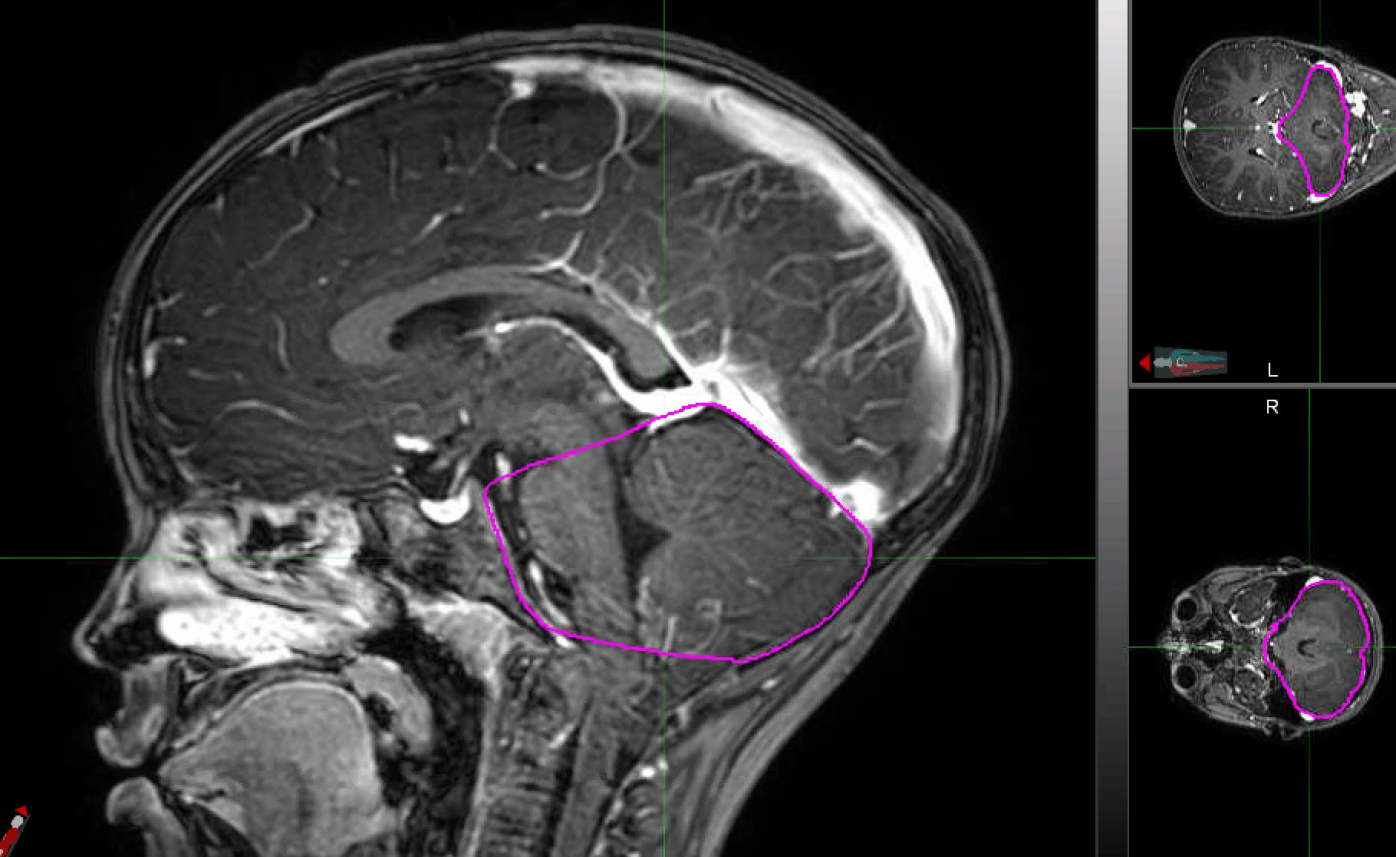
Posterior Fossa Contour in MIM®

Approximations and calculated estimates of the region of interest volume exist to more exact contemporary methods, namely manual segmentation via software platforms such as 3D-Slicer (www.slicer.org.) [11]. Unfortunately, manual segmentation presents clinicians and researchers with significant challenges [11, 12]. Proper segmentation relies upon the user’s knowledge of the anatomy, precision, time, and effort, taking up to several hours for a complete and accurate contour (Fig. 2). Medical physicists, radiation oncologists, and neurological surgeons alike have been required to devote significant time to manual segmentation, as it is an integral component of radiotherapy treatment plans. Furthermore, inter-operator variability and even intra-operator variability may threaten the results of even expert radiologists due to the subjective nature of contouring [11–13].

Significant progress has been made in the field of segmentation in recent decades. Semi-automated segmentation, while still requiring user input, algorithmically contributes to the contouring process. Recent years have also seen the advent of more sophisticated fully automated segmentation techniques. Open-source semi-automated and automated segmentation software does not require the user to possess computer programming skills or mathematical knowledge and demands less overall effort on the part of the user. A more facile segmentation process enables the clinician and researcher to explore conditions for which a comprehensive understanding of their pathophysiology of interest is still incomplete [11–14].

Despite the potential advantage a common approach to posterior fossa segmentation would present, a universal technique or software enabling this does not exist. Yet, for the dual purpose of validating the results of, and enhancing the reproducibility of studies into the posterior fossa, a consensus on segmentation methodologies can be reached, to guide work in this field in the future. The purpose of this review is to provide researchers and clinicians alike with an objective understanding of the segmentation techniques, software applications, and algorithms that have been specifically used in the context of the posterior fossa. To our knowledge, this is the first systematic review to explore the usage of segmentation in this location, in which the nuanced anatomy plays a key role in several pathological processes.

## Materials and methods

### Object

This systematic review summarized and investigated all of the literature related to various segmentation techniques in the setting of the posterior fossa was first performed by the Preferred Reporting Items for Systematic Reviews and Meta-analyses (PRISMA) guidelines [15].

### Research strategy

Systematic literature searches were conducted by a medical librarian (AM) in the electronic databases PubMed/MEDLINE (through October 2023), Embase (through October 2023), Cochrane Library (through October 2023), and Web of Science (through October 2023) using both controlled vocabulary terms and text words. The searches were performed without any geographical limitation but included only English language publications. The search of databases was conducted using the specific medical subject heading (MeSH) in the search were “Volumetric Segmentation” OR “Volumetry” OR “Segmentation” AND “Posterior fossa volume” OR “Fossa volume” OR “Volume Fossa”.

**Fig. 3:**
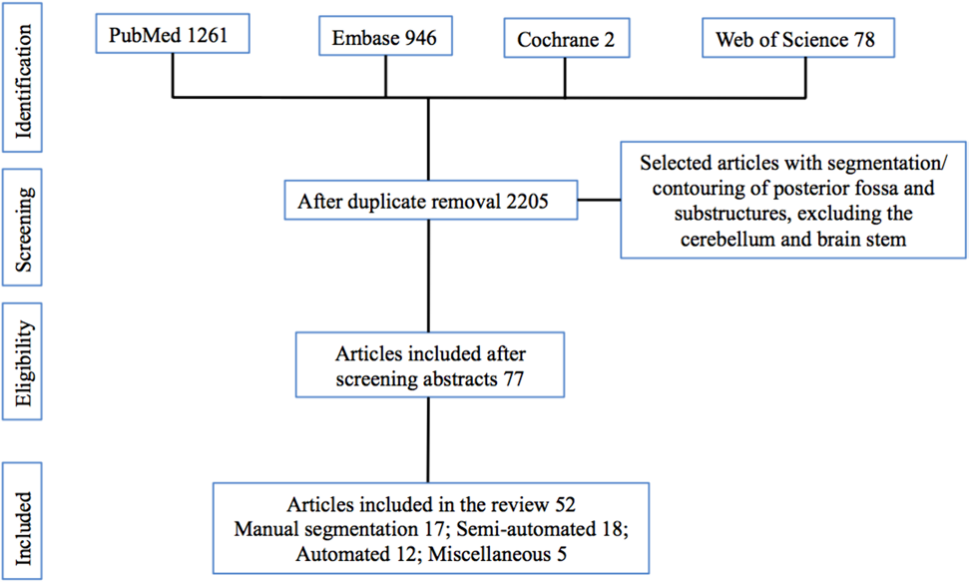
PRISMA diagram

All references were imported into Endnote reference software and de-deduplication was performed. They underwent further de-duplication after screening by a team of reviewers and then joint articles. All studies including case reports, letters to editors, review articles, and other relevant data were enrolled in the study. The titles and abstracts of all articles attained from the databases were reviewed by two senior independent authors to investigate eligibility based on the aim of the study. Then, full texts of the related articles were enrolled; duplicated and non-related articles were excluded. Finally, three clinical studies were enrolled and reviewed (Fig. 3).

### Inclusion and exclusion criteria

The inclusion criteria in the present study were, all clinical studies in English up to October 2023 that investigated the segmentation techniques, software applications, and algorithms that have been specifically used in the context of the posterior fossa, English studies, and studies on humans. The two reviewers searched the databases mentioned before based on the title and abstract of the studies. On the other hand, the papers on animal models and non-English articles were excluded from the study.

## Study outcomes

The primary outcome of this review was to investigate the various segmentation techniques, and their respective strengths and weaknesses, employed along with the objectives and outcomes of the various studies reported in the literature.

## Results

The literature search identified a total of 2205 articles. After screening abstracts, titles, and methods sections, 77 articles discussed the topic of interest fulfilling the eligibility requirements for full-text review. Publications withdrew (1 article), based on phantom segmentation (2 articles), and relevance (22 articles) were eliminated leaving 52 papers for inclusion in the review article.

The most commonly used segmentation technique was semi-automated segmentation (18 articles). Manual segmentation was the second most common technique (17 articles). Twelve articles utilized automated segmentation techniques, and 5 articles used miscellaneous techniques that included the Cavalieri estimator, a point-counting method that uses a grid of points to estimate the volume of a region and a technique based on linear measurement formulas.

Of the 52 articles the most commonly addressed pathologies identified in our literature search were Chiari malformation (14 articles), and neurovascular compression syndromes, including trigeminal neuralgia and hemifacial spasm (8 articles). Other articles about craniosynostosis (3 articles), multiple sclerosis (4 articles), and Research related to other neuropathological conditions involving posterior fossa malformations and anomalies including segmentation of tumors and multiple sclerotic lesions as well as studies of the posterior fossa volumetric changes in Dandy-Walker malformations (DWM), hypertrophic olivary degeneration, post-operative pediatric cerebellar mutism, hemifacial spasms, Crouzon syndrome, craniosynostosis, achondroplasia, trigeminal neuralgia and other neurovascular compression syndromes.

### Various volumetric segmentation methods

#### Manual segmentation approaches

Though capable of producing the most accurate results, manual segmentation is tedious and extremely time-intensive. Nonetheless, the literature search revealed numerous publications describing manual segmentation to measure posterior fossa volume for a variety of neurological conditions (Table 1) [16–32].

**Table 1:** Manual segmentation of the posterior fossa: summary of studies, imaging modalities, segmentation software, and techniques

Ref	Study Type	Patients/Controls	Imaging, Methods, Segmentation Techniques and Software	Time/ computational power details	Segmentation/Study Significance and Outcome	Operated and supervised by
Cheng et al. [16]	PF^a^ crowdedness in hemifacial spasms	102/51	T1- and T2-weighted MRI, 3D-T2-DRIVE. 3D-slicer	3.0 T MRI, TR: 2000 ms, TE: 200 ms, thickness, 0.5 mm	Patients with hemifacial spasms had greater PFCI^b^ and lower CSF volumes	Radiologist
Cheng et al. [17]	Association of Primary TN^c^ with PF crowdedness	46/46	T1- and T2-weighted MRI, 3D-T2-DRIVE. 3D-slicer	3.0 T MRI, TR: 4500 ms, TE: 12 ms/96 ms, thickness, 2 mm	PFCI is associated with a disease state. Test–retest reliability Pearson correlation coefficient = 0.83, p < 0.001	Radiologist
Chadha et al. [18]	PF: linear dimensions and volumetry	16/31	T1-weighted MRI. Fiji-(Image J)	1.5 T MRI, TE = 5.47, TR = 12, thickness, 1 mm	PFVs^d^ are greater in men, and not affected by supratentorial tumors	Neurosurgeon
Pellerin et al. [19]	Crouzon syndrome: PFV studies	41/70	CT. Freeware Horos V 3.3.6 (https://horosproject.org)	-	PFV in Crouzon syndrome is heterogeneous; (30 patients’ volume < controls; 10 patients > controls)	Neurosurgeon
Milarachi et al. [20]	PFV in IIH ^e^	27/14	MRI	-	PFV and PFV/ICV^f^ of control ~ patients	-
Roller et al. [21]	PF size in adult CM-I vs. controls	28, 21/113	3D GRE T1-weighted MRI. AW Suite software (version 2.0, GE Healthcare)	3.0 T or 1.5 T MRI, TR/TE of 190/2–3, thickness, 1 mm	Linear measurements of PF do not accurately predict PFVs. No difference between PFV and PFV/ICV was seen. Intra- and Interobserver reliability = 0.98 and 0.99, respectively	neuroradiologist
Taylor et al. [22]	CM-I patients with crowded vs. uncrowded PF	45/-	T1-weighted MRI. OsiriX software	––	PF crowdedness alone does not explain CM-I pathogenesis (p = 0.33). Intra- and inter-observer reliability = 0.98 and 0.99, respectively	Neurosurgery resident
Noudel et al. [23]	PFV increase post CM-I surgery	11/-	T1-weighted MRI. Advantage Windows Volume Analysis software (version 4.3)	1.5 T MRI, thickness, 2.5 mm	Increased PFV is associated with the improvement of symptoms	Experienced observer
Krishna et al. [24]	Diffusion tensor imaging assessment of microstructural brainstem integrity in CM-I	8/16	T1-weighted, DTI co-registration. 3D Slicer	3.0 T MRI, TE 5 ms; TR 12 ms, thickness, 3 mm	Microstructural alterations associated with CM-I diagnosis. Elevated fractional anisotropy in the lower brainstem seen vs. controls	Neurosurgeon, Radiologist
Seaman et al. [25]	Fourth ventricle size change in CM-I	72/30	Axial T2-weighted MRI. Vitrea software (Vital Imaging version 6.7, Minnetonka, Minnesota, USA)	––-	Enlargement of the fourth ventricle in CM-I is associated with worse clinical presentation (P = 0.0002), independent of PFV (P = 0.2412)	Neurosurgeon
Payabvash et al. [26]	Differentiation of PF tumors involving the fourth ventricle	-/74	T2-weighted MRI, Postcontrast T1-weighted MRI, FLAIR, ADC/DWI. GE Advantage Workstation	1.5 -3.0 T MRI, TE = 107, TR = 3600, thickness, 2 mm	Quantitative voxelwise ADC^g^ histogram analysis helps differentiate PF tumors; 10th percentile ADC values with ROC AUC of 0.877 for identifying medulloblastoma	neuroradiologist
Li et al. [27]	Preoperative differentiation between ependymoma and pilocytic astrocytoma using a radionics approach based on machine learning	45/-	T1- and T2-weighted MRI. Image J software (NIH), MATLAB	3.0 T MRI, thickness, 1 mm	Efficient approach for pediatric PF tumor differentiation. High accuracy (0.80) is seen with texture features vs. Gabor transform (0.67) and wavelet transform (0.73) based features	Radiologist
Gutierrez et al. [28]	Differentiate histologic tumor types in PF by using support vector machine classifiers on quantitative MRI features	40/-	Contrast-enhanced T2- and T1-weighted MRI. NeuROI (http://www.nottingham.ac.uk/research/groups/clinicalneurology/neuroi.aspx), MATLAB	1.5–3 T MRI, TR = 4883–5800 ms, TE = 59–89 ms, thickness, 0.5–2.5 mm	Correct differentiation of pediatric PF tumor types (> 91.4%) was achieved via support vector machine-based classifiers using apparent diffusion coefficient (ADC) histogram features	Radiologist and trained clinical research fellows
Jin et al. [29]	Gait characteristics and clinical relevance of hereditary spinocerebellar ataxia on deep learning	30/23	T1-weighted MRI (3D-MP-RAGE). NiftyReg, MITK, MATLAB	3.0 T MRI, TR = 2200 ms, TE = 2.48 ms, slice, thickness, 1 mm	Gait parameters, ataxia scales, and midsagittal cerebellar proportion in the posterior fossa may be used as clinical markers for spinocerebellar ataxia	Neuroradiologist
Rosenblum et al. [30]	To investigate the effect of endothelial PAS domain-containing protein 1 mutation on PF development	8/-	T1- weighted sagittal MRI. OsiriX software	-	Incidental PF malformations were found in patients with the mutation, not associated with a small PF	–––––––-
Sgouros et al. [31]	2- and 3-D measures of vestibular schwannomas in PF	58/-	T2-weighted CISS MRI. GammaPlan workstation	–––––-	Maximum and transverse diameters accurately predict tumor volume and brain shift, respectively. Vestibular schwannoma volume > 2 cc (ROC 0.963), and a 14.5 mm cerebellopontine angle transverse diameter (ROC 0.979) is linked to brain shift	–––––- –––––
Thatikunta et al. [32]	Volumetric changes in posterior vault distraction in lambdoid craniosynostosis	11/-	CT. 3D Slicer software	1.5 T MRI, TR/TE 12.2/5.9 ms, thickness, 0.4 mm	38.7% increase in PFV and 26.9% increase in foramen magnum area observed postoperatively	-

^a^PF = Posterior fossa

^b^PFCI = Posterior fossa crowdedness index

^c^TN = Trigeminal neuralgia

^d^PFV = Posterior fossa volume

^e^IIH = Idiopathic intracranial hypertension

^f^ICV = total intracranial volume

^g^ADC = Apparent diffusion coefficient

Cheng et al. explored the relationship between the hemifacial spasm and posterior fossa crowdedness index, defined as the ratio of hindbrain or cerebellar tissue volume to PFV [16].

CSF volume was also measured and defined as the space between the posterior fossa and hindbrain outlines. Volumes were measured by a radiologist via manual segmentation using 3D-Slicer software. Patients with hemifacial spasms were found to have greater crowdedness indices and lower CSF volumes. Lower crowdedness index was significantly associated with superior short-term outcomes, defined as the absence of twitching within 7 days of microvascular decompression. Long-term outcomes, determined by a follow-up period of at least 2 years, were not significantly associated with differences in the crowdedness index. In a separate study, Cheng et al. also examined posterior fossa crowdedness in the context of trigeminal neuralgia [17].

A radiologist manually segmented both the posterior fossa and the brain tissue within the fossa area, which included the fourth ventricle, to serve as the PFV and hindbrain volume respectively. The ratio of hindbrain volume to PFV served as the posterior fossa crowdedness index, which was found to be greater in patients with trigeminal neuralgia. Furthermore, both women and younger individuals were more likely to possess more crowded fossae. Test–retest reliability, calculated by having the same radiologist repeat the measurements one week later, was found to be high with a Pearson correlation coefficient of 0.83. Another study utilized manual segmentation to find the PFVs of patients with supratentorial tumors [18] using the Fiji open-source software’s [33] region of interest manager to delineate boundaries. Linear measurement-based formulas were used to approximate the PFV. Researchers calculated the volume of the region by assuming the posterior fossa was an inverted cone truncated at the foramen magnum. The radius of the cone’s base was half the distance from the dorsum sellae to the inion. The height of the truncated cone was the distance between the line segment of the seller to the inion and the line segment of the basion to the opisthion. Thales’ theorem was used to calculate the height of the cone segment lying below the foramen magnum to ultimately determine the volume of the truncated cone. Researchers also assumed that the posterior fossa was an ellipsoid. Volume was equal to half of the product of the basion-tentorium apex distance, apex-inion distance, and transverse diameter. The transverse diameter was the sum of the total transverse slice thickness and interslice distance. Manual segmentation was far more accurate than either of these methods. The ellipsoid approximation possessed a higher agreement index than the inverted cone method and was deemed to be more concordant with the results of manual segmentation.

Pellerin et al. compared the intracranial volumes and PFVs of patients with Crouzon syndrome to those of age-matched controls [19]. Freeware Horos V 3.3.6. (https://horosproject.org) was used for manual segmentation. The closed polygon tool was first applied to every tenth slice, followed by interpolation of the intervening slices. This region of interest was edited slice by slice using the repulsor tool. This process was labor-intensive, requiring approximately one hour per study for a sufficiently trained researcher. The study’s results suggest that PFV in Crouzon syndrome is heterogeneous with 30 out of 41 patients having smaller fossae and 10 having larger PFVs than their respective controls.

In a study conducted by Milarachi et al., manual segmentation was used to compare the PFVs of subjects with intracranial hypertension to those of matched controls [20]. Though initially no significant difference in PFV was found, multivariate logistic regression analysis demonstrated a decreased likelihood of intracranial hypertension with a relative increase in PFV.

Another study used AW Suite software (version 2.0, GE Healthcare) to manually segment the PFVs of adult CM-I patients and controls [21]. While no significant difference was found in PFV and the ratio of PFV to intracranial volume, male sex, white race, and increased BMI were significantly associated with larger PFVs. Intra- and Interobserver reliability, which were 0.98 and 0.99 respectively, were assessed by having the original reader as well as a second reader repeat the measurements for 25 randomly selected patients.

A study by Taylor et al. used open-source OsiriX Imaging Software [34, 35] to evaluate the PFVs of CM-I patients [22]. They categorized patients into either the crowded posterior fossa group or the spacious group based on midline sagittal T1-weighted images. Subsequently, all relevant structures were manually contoured. The PFVs and posterior fossa tissue content volume did not differ significantly between crowded and spacious fossa patients. The ratio of posterior fossa tissue content volume to PFV was, however, significantly greater for crowded fossa patients compared to spacious fossa patients. Even though a greater proportion of crowded fossa patients had syringomyelia, which has previously been observed to be associated with smaller PFVs, the difference was not significant. Intra- and inter-observer reliability, which were 0.98 and 0.99 respectively, were assessed by having the original reader as well as a second reader repeat the measurements for 25 randomly selected patients.

Noudel et al. measured the change in PFV following craniectomy in 11 patients with symptomatic CM-I [23]. The study demonstrated a significant association between increased PFV and symptomatic improvement. Axial T1-weighted MR images with a 1-mm intersection gap and 2.5 mm slice thickness were reshaped into approximately 70 equally spaced oblique slices parallel to the nasion-basion line by the algorithm used. The posterior fossa was determined to be limited rostrally by both the top of the tentorium and the midbrain-pons junction. The anterior cranial boundary was the ventral point of the midbrain-pons junction to produce more measurable segmentations compared to including the midbrain by using the diencephalon-midbrain junction. Due to the elimination of the opisthion during surgical decompression, the caudal limit of the posterior fossa was defined by the nasion-basion line, which transected the brainstem. Manual contouring of each reshaped slice was performed, and volume was subsequently calculated by first finding the product of the region of interest surface area and the slice thickness for each slice. Following this step, the volumes of all of the slices as well as the inter-slice gaps were added together to determine the PFV. This technique relied heavily upon manual segmentation but enabled the researcher to contour slices that were parallel to the nasion-basion line. Thus, the algorithm better defined the boundaries of the fossa and yet did not facilitate the actual act of contouring. An overview of additional studies utilizing manual segmentation of posterior fossa in CM-I patients investigating the integrity of microstructural brainstem as well as change in fourth ventricle size is described in Table 1 [24, 25].

Tumor management research and treatment planning have also employed manual segmentation. Payabvash et al. utilized quantitative voxelwise apparent diffusion coefficient (ADC) histogram analysis to differentiate posterior fossa tumors, with up to 82% and 95% sensitivity and specificity, respectively [26]. Li et al. utilized a radiomics approach based on machine learning to preoperatively differentiate pediatric posterior fossa tumors [27]. The authors report higher accuracy with texture features versus Gabor and wavelet transform-based features. Gutierrez et al. have also reported the differentiation of pediatric posterior fossa tumor types via support vector machine-based classifiers using apparent diffusion coefficient (ADC) histogram features [28]. (Table 1).

Jin et al. studied gait characteristics of hereditary spinocerebellar ataxia patients to examine the correlation between gait parameters, clinical scales, and imaging. The study found that gait parameters, ataxia scales, and midsagittal cerebellar proportion in the posterior fossa may be used as clinical markers for spinocerebellar ataxia [29].

#### Semi-automated segmentation approaches

Table 2 shows semi-automated segmentation of the posterior fossa: summary of studies, Imaging modalities, segmentation software, and techniques in different articles enrolled in the study [28–53].

**Table 2:** Semi-automated segmentation of the posterior fossa: summary of studies, Imaging modalities, segmentation software and techniques

Ref	Study Type	Patients/Controls	Imaging, Methods, Segmentation Techniques and Software^1^	Time/ computational power details	Segmentation/Study Significance and Outcome	Operated and supervised by
Spiteri et al. [36]	ION^1^ volume and the development of PF syndrome	28/-	T2-weighetd MRI. Seed growing	3.0 T MRI, TE: 12 ms/96 ms; TR: 4,500, thickness, 2 mm	Left ION hyperintensity by MRI correlates with PF syndrome (average AUC 0.88, % accuracy 84.52). First semi-automated segmentation study to quantitatively confirm the correlation of HOD^1^ with PF syndrome	Neuroradiologist
Avula et al. [37]	Association of POPCMS^1^ with HOD	28/-	T2-weighetd MRI. Seed growing	1.5 T MRI, TE: 110 ms; TR: 4924, thickness, 5 mm 3.0 T MRI, TE: 12 ms/96 ms; TR: 4,500, thickness, 4 mm	Quantitatively confirmed association of POPCMS with bilateral HOD. Hyperintensity in the left ION is a reliable marker of POPCMS (AUC 0.9, 0.71, 0.91)	Radiologist
Sgouros et al. [38]	PFV and CM-I	42/51	T2- weighetd MRI. Seed growing	–––-	PFVs of CM-I patients are not smaller than controls (p = 0.036), but those with syringomyelia and CM-I are (p = 0.004)	––––––
Tanrikulu et al. [39]	Visualization of cranial nerves and vessels in PF	20/-	T2-weighted MRI_CISS_. Volume growing	1.5 T MRI, TR/TE 12.2/5.9ms, thickness, 0.4 mm	Successful identification of compressed nerves. Trigeminal nerve compression sensitivity 0.97, specificity 0.90	–––––––
Naraghi et al. [40]	3D visualization of neurovascular relationships in PF	55/-	T2-weighted MRI_CISS_. Volume growing. SegMed (segmentation), Qvis (volume rendering)	1.5 T MRI, TR = 6490 ms, TE = 98 ms, thickness, 0.7 mm	Pre-surgical segmentation data correspond to surgical findings	Neurosurgeon
Vatansever et al. [41]	Compare PF growth trajectories during pregnancy for controls and fetuses with cerebellar malformations	53/79	T2-, T1-weighted MRI. ITK-SNAP	1.5 T MRI, TR = 1500 ms, TE = 160 ms, thickness, 2.5 mm	Demonstrated advantage of 3D visualization as volume changes not seen in 2D; Dice coefficient inter-rater variability (0.90) and intra-rater variability (0.95)	Neuroradiologist
Horínek et al. [42]	Role of anatomical configuration of PF and its substructures in the occurrence of neurovascular conflict in TN	18/15	MRI (axial 3D-FIESTA). ITK-SNAP	TR = 1000 ms, TE = 139 ms, thickness, 0.5 mm	Study failed to show correlation between clinical neurovascular conflict and the size of PF or its substructures. Intraindividual variability for PFV measurement = 1%	Neuroradiologist
Khalsa et al. [43]	PFV and clinical outcome after CM-I decompression	42/-	T2-weighted MRI. Edge detection, Intensity thresholding. MATLAB	–––––	Increased post-surgery PFV of 7.74% is associated with improved headache symptoms vs. 2.26% increase with no improvement (p = 0.015)	Operator
Khalsa et al. [44]	PFVs of symptomatic vs asymptomatic CM-I patients	51/51	T2-weighted MRI. Edge detection, Intensity thresholding. MATLAB	–––––-	No significant difference in PFV (183.4 vs. 190.6 mL; p = 0.250) between symptomatic and asymptomatic CM-I patients; also no difference in PFCI	Neurosurgeon
Gawne-Cain et al. [45]	Lesion volume in multiple sclerosis vs. disability: MRI sequences comparison	25/-	CSE-, FSE-, fFLAIR-MRI. Local thresholding	1.5 T	PF lesion volumes were greater for CSE; Significant correlation seen between total lesion volume and Kurtzke extended disability scale with both sequences (CSE, r = 0.49; fFLAIR, r = 0.44)	Neurologist
Chan et al. [46]	Small PF CSF space as a risk factor for hemifacial spasm	41/41	T2-weighted MRI_CISS_. Thresholding. Analyze 8.1 software	–––-	Hemifacial spasm patients had lower CSF volumes than controls (p = 0.007)	–––––––
Mantha et al. [47]	CSF space change vs*.* outcome post CM-I decompression	57, 36/-	T1-weighted MRI. Thresholding, OsiriX software	–––––––	CSF volume changes did not correlate with the age-related differences in clinical outcomes. Inter-observer measurement error ~ 5 percent	Neurosurgeon
Lirng et al. [48]	PFCI^o^ is associated with age and sex	-/52	3D fast SPGR MRI. Thresholding	1.5 T, TR/TE/NEX of 500/minimal/2, thickness = 5 mm	Men had greater PFVs, and women had greater PFCIs, which declined with age. Test–retest reliability correlation coefficient = 0.99	
Patibandla et al. [49]	Volumetric analysis/long-term outcomes of stereotactic radiosurgery for benign WHO grade-I PF meningiomas	120/-	T1-weighted MRI. Numerical integration via the trapezoidal rule. GammaPlan or NIH ImageJ software	––––	Tumor volumetric change 3 years post-surgery predicted tumor burden up to 10 years; 5 years R^2^ = 0.756, p < 0.001; 10 years R^2^ = 0.421, p = 0.001; 5–10-year tumor response R^2^ = 0.636, p < 0.001	Attending neurosurgeon and neuroradiologis
Jure et al. [50]	Assess gray matter injury variability in early MS	18/24	Localizer scout, transverse FSE proton density-, T2-, and transverse proton density-weighted spoiled GRE. Semi-automated segmentation of lesions	1.5 T, TR/TE1/TE2 of 2600/15/85, thickness = 3 mm	Analysis of brain magnetization transfer maps showed large gray matter injury variability in the early stages of MS	––––
Manava et al. [51]	Neurovascular compression at the ventrolateral medulla in patients with arterial hypertension	44/-	3D-CISS and 3D-TOF MRI. Volume growing. Fusion with TOF data (manual labeling)	3.0 T, CISS: TR 7.48 ms, TE 3.23 ms, TOF: TR 21 ms, TE 3.77 ms, thickness = 3 mm	Significantly improved representation with fused data seen for sum of all vessels (p < 0.001); The method outlines the anatomical course of vessels and nerves in the posterior fossa	Neurosurgeon and neuroradiologist
Hastreiter et al. [52]	Optimized representation of neurovascular relationships in PF	80/-	3D-CISS and 3D-TOF MRI. Volume growing. Data fused post registration/segmentation	–––––-	Significantly improved representation with fused data; Using a 0–5 scoring, a score of 3.96 (SD 1.59) for 3D fused data vs. 2.65 (SD 1.77) for 3D-CISS	Neurosurgeon
Pitsika et al. [53]	Arachnoid cysts volume changes post-fenestration in children	4/-	T2-weighted MRI. 3D Slicer/semi-manual	––––––-	30% reduction in posterior fossa cyst volume	

### Seed growing/volume growing segmentation

Several studies utilized “seed growing” otherwise known as volume growing segmentation. Region or seed growing is a technique in which seeds or pixels/voxels are selected either by the user or by the algorithm [54]. A region of interest spreads outward from these seeds and neighboring voxels are added to the region according to intensity similarity. Noise may interfere with the addition of voxels and the overall quality of the segmentation depends on the initial seed selection.

Spiteri et al. used “seed growing” to investigate the relationship between inferior olivary nuclei (ION) volume and the development of posterior fossa syndrome [36]. The seed point was chosen manually using anatomical knowledge. The region growing outward from this point incorporated voxels with a certain intensity within a four mm radius. Moreover, voxels with a gray-level intensity lying within a threshold-determined range of the intensity of the seed point were selected. However, the differential in intensity between any voxel and all adjacent voxels was also dependent upon a set threshold. These processes were repeated until the region of interest remained constant. The entire segmentation process was repeated three times for each set of imaging. These same authors utilized the aforementioned “seed growing” technique to demonstrate a significant association between bilateral hypertrophic olivary degeneration and pediatric cerebellar mutism following posterior fossa tumor resection in another study [37]. A neuroradiologist validated and edited the results of segmentation (Table 2).

The same technique has also been used by researchers to examine the relationship between posterior fossa volume (PFV) and CM-I [9]. These algorithms were supplemented with manual segmentation to cover certain anatomic areas, including the midbrain-pons junction and cerebellar-cerebral junction. As described by the authors in a previous study using the same approach, T2-weighted imaging was deemed necessary given the fact that only adjacent voxels with similar shades of gray were incorporated into the region of interest [38]. The authors used an X-Windows-based C language program on a UNIX operating system workstation. Intracranial volume (ICV) was found as well as the ratio of PFV to ICV to account for differences in head circumference. The resulting volumes and ratios of the Chiari group were compared to those of a control group.

Unfortunately, the results of seed-growing segmentation may be skewed due to dependency on the selected seed-point, and by selecting a different seed-point a researcher can achieve a different segmentation result [54]. Furthermore, the threshold used to grow the region of interest is a critical safeguard against accidentally incorporating external structures. Additionally, the region of interest must be homogenous as segmentation performance is significantly impaired by noise due to the introduction of heterogeneity. A study conducted by Tanrikulu et al. aimed to perform a virtual endoscopy of the cranial nerves and vessels within the brainstem in patients with trigeminal neuralgia [39]. Anisotropic diffusion was first used to decrease noise, thereby increasing homogeneity, and allowing for greater ease of intensity-based segmentation. Automatic volume growing was used to segment the CSF space, followed by manual segmentation of the cranial nerves to generate a 3D representation of the posterior fossa region. The segmented voxels were then assigned color and opacity values, rendering CSF transparent and the vessels and nerves opaque. A resident and attending physician reviewed the virtual space and agreed on the vessel that was compressing the nerve 20 out of 20 times. The sensitivity of neurovascular compression was 0.97 and the specificity was 0.90.

Another study used similar techniques to visualize the cranial nerves in the posterior fossa of 55 patients with neurovascular compression symptoms [40]. Noise reduction was performed via anisotropic diffusion and morphological filtering was used to remove hypointense signals within CSF. Volume-growing segmentation was accomplished using user-defined bounding boxes and a specified threshold range. The resulting volume served as a mask that was subsequently used to label the structures. Semi-automated segmentation allowed the CSF containing cranial nerves and vessels and the brainstem to be separated from the surrounding region. Manual labeling of the vessels and cranial nerves was required due to a similarity in intensity between the two types of structures. Brainstem segmentation was performed via volume growing. The accuracy of these 3-dimensional visualizations was demonstrated intraoperatively, and the aforementioned steps took approximately 2 to 4 h to complete. Segmentation was accomplished via the Linux-based software SegMed [52]. This technology allows a clinician a greater understanding of the anatomy of neurovascular compression. This technology is sensitive to noise, and if MR CISS (T2-constructive interference in steady state) sequences are used exclusively, it is impossible to differentiate cranial nerves from vessels based on intensity, which are both hypointense within the hyperintense CSF. Fortunately, anatomical knowledge may be used to overcome this challenge.

Manava et al. [51] studied neurovascular compression at the ventrolateral medulla in patients with arterial hypertension. Volume growing was performed to segment both the brainstem and the surrounding CSF. Cranially nerves were manually segmented. The approach also consisted of volume-growing segmentation of the vessels in three-dimensional time-of-flight (3D-TOF) angiography. Registration and fusion of the CISS data with 3D-TOF was accomplished. The team demonstrated that incorporating vascular information from TOF into CISS can improve 3D visualization by reducing artifacts in CISS data. Significantly improved 3D visualization with fused data in the context of posterior fossa has also been demonstrated by Hastreiter et al. [55]. Using a scoring system that ranged from 0 to 5, to increase visualization accuracy, it was determined that fusion significantly improved model accuracy.

### ITK-SNAP (Active Contour Evolution)

ITK-SNAP (http://itksnap.org) is an open-source tool designed for visualization and manual and semi-automatic segmentation of 3D medical imaging. To assess posterior fossa development during the second and third trimesters, Vatansever et al. carried out semi-automatic segmentation via ITK-SNAP [41]. T2- and T1-weighed as well as SNAPIR sequences of 53 fetuses with posterior fossa abnormalities and 79 controls were used. Segmentation was performed by a single rater, assisted by 2 fetal neuroradiologists. Preprocessing entailed the selection of an intensity threshold by the rater. Following this step, the rater would place “seeds” or “bubbles” within the region of interest. A user interface allows the rater to select parameters for contour evolution, including the weights of terms or forces that affect the evolution of the contour [27]. The rater was able to halt or rewind this contour evolution. Ultimately, the rater was able to manually edit the area. The mean Dice coefficients for inter- and intra-rater variability were 0.90 and 0.95 respectively. Such a method greatly enhances the user’s control over the contouring process, enabling him or her to fine-tune the segmentation process and even reverse it. The user interface also explains how changing each parameter or weight affects the evolution of the contour. Employing ITK-SNAP and semi-automated segmentation, Horínek et al. [42] investigated the role of the anatomical configuration of the posterior fossa and its substructures in the occurrence of neurovascular conflict in trigeminal neuralgia. However, the study failed to show the correlation between clinical neurovascular conflict and the size of the posterior fossa or its substructures.

### Edge detection

Automated edge detection was employed by two studies, both of which investigated pediatric cases of Chiari malformation Type I. Automated edge detection is a common form of segmentation in which edges are defined as boundaries where intensity changes sharply [54]. One study by Khalsa et al. utilized a MATLAB-generated program in which axial T2-weighted MRI sequences were inputted into a graphical user interface [43].

These DICOM files were reconstructed into a midsagittal view to allow the user to select vertices of the posterior fossa. The superior vertices of the tentorium and clivus were used to define the clivus-tentorium border. A Gaussian filter was used to smooth the imaging and remove noise, thereby enabling the program to accurately locate local maxima in the intensity gradient on each axial slice to serve as an edge. Following this step, the user either manually modified the contour or clicked within the bounded area to produce a binary mask, where the posterior fossa voxels have a value of 1 and other tissue voxels have a value of 0. This process was repeated for all posterior fossa axial slices, with the final volume stored as a 3D binary matrix. The researchers found that increased PFV following decompression was significantly associated with symptomatic improvement. This technique, unfortunately, was not generated by open-source software and required programming. By allowing the user to select the vertices of the clivus-tentorium border the program increased the accuracy of the anterior cranial boundary, which was further improved by an edge-detection process with optional manual editing.

A second study conducted by the same research group utilized a similar custom semi-automated tool to determine whether decreased PFV was associated with Chiari malformation type I symptoms [44]. Out of 102 pediatric patients, only 78 had recorded PFVs due in part to poor axial image quality interfering with proper edge detection. No significant difference in PFV was found between symptomatic and asymptomatic patients.

### Thresholding

Thresholding is a segmentation technique in which a voxel intensity histogram is used to find intensity values that may be used to partition voxels into different tissue classes. All voxels with intensities greater than a specific value are classified as a particular tissue type and all voxels with intensities less than or equal to the specified value are classified as an alternate tissue type or perhaps background. In the case of global thresholding, only a single threshold is used but in the case of local thresholding, the threshold that is used depends upon the position in the image [54]. The search revealed 6 papers that used thresholding-based techniques.

A study by Gawne-Cain et al. utilized local threshold segmentation to measure the volume of multiple sclerosis lesions, including posterior fossa lesions [45]. Three sequences of imaging, CSE, fast spin echo, and fFLAIR, were used. Researchers performed segmentation twice, with the second round preceded by anisotropic diffusion. Segmentation times were dramatically reduced with fFLAIR and FSE images to 4–30 min versus 45–60 min seen in the case of CSE images.

Another study that used intensity threshold segmentation analyzed whether patients with hemifacial spasm have lower posterior fossa CSF volumes than matched controls [46]. The commercial software Analyze 8.1’s threshold tool within the region of interest module was first used on posterior fossa-focused T2-weighted MRI CISS sequence. Subsequently, these segmentations underwent manual editing. Researchers determined that the volume of CSF was significantly lower (11.4%) in patients with hemifacial spasms compared to controls.

In the aforementioned study by Khalsa et al. (see edge detection section) researchers used thresholding to analyze the impact CSF volume change has on Chiari malformation type I symptomatology following surgical decompression [43]. After segmenting the posterior fossa, the user-outlined the region of interest with a polygon. Thresholding was performed, selecting the hyperintense CSF voxels. The effects of noise were mitigated by removing voxels with fewer than a specified number of connected voxels according to the 8-voxel-connected neighborhood of each selected voxel. Another study, which was also described previously in the edge detection Sect. [44], utilized the same CSF subvolume segmentation technique. Researchers failed to find any significant difference in the ratio of CSF volume to caudal PFV (crowdedness index) between symptomatic and asymptomatic Chiari patients.

A study by Mantha et al. used thresholding to analyze the effects of CSF volumetric change on post-operative Chiari malformation outcomes [47]. OsiriX software was utilized for volumetric measurements on T1-weighted imaging. OsiriX, an open-source software running only on Macintosh products, has a thresholding tool that can be used for segmentation purposes. The user selects the voxel of interest and chooses the upper and lower limits of the voxel intensity of added voxels. Users also may view the outcome of segmentation before the results are finalized. In this study, a user manually contoured the CSF spaces within the posterior fossa on axial sequences, overestimating the region. Thresholding was then used to select the CSF. Though a single party, the first author, performed all measurements, inter-observer measurement error was found to be approximately 5 percent. It is noteworthy that OsiriX grants users the ability to both manually contour regions of interest as well as generate a region of interest based on signal intensity.

A study reported by Lirng et al. utilized a semi-automated ad hoc technique that required a neuroradiologist to manually pre-segment the posterior fossa on axial 3D fast SPGR images [48].

Researchers wrote the software in interactive data language. A histogram of signal intensities contained two peaks, each of which represented either CSF or hindbrain voxels. A “cutting point” served as the intensity level that divided the low-intensity CSF from the high-intensity hindbrain. All voxels below a certain intensity level were excluded as air, and all voxels above a certain intensity level were excluded as vessels. The posterior fossa crowdedness index was defined as the ratio of hindbrain volume to PFV. The same neuroradiologist re-segmented 10 patients’ posterior fossa to assess test–retest reliability, which was found to be high with a correlation coefficient of 0.99. Men were found to have greater PFVs as well as hindbrain volumes. However, the crowdedness index was greater for women than men and was inversely correlated with age.

### Automated segmentation approches

The search retrieved 12 publications [3, 56–66], that utilized various automated segmentation techniques that included atlas-based segmentation and techniques utilizing an algorithm that simulates neurons in the brain referred to as “neural networks”. An atlas or template possesses anatomical information and is used as a reference for the segmentation of a particular structure in provided imaging [54]. To utilize an atlas to segment a region of interest, it must first be aligned with imaging, a process known as registration. Affine registration is the traditional alignment method, though it may be inadequate if the average atlas anatomy differs significantly from that of the target imaging due to inter-subject heterogeneity. In such cases, non-linear registration is required.

A study conducted by Bagci et al. employed open-source atlas-based automated segmentation to determine the PFVs and hindbrain volumes of Chiari malformation Type I patients and healthy controls [3].

The atlas was constructed from the T1-weighted images of Chiari patients that had been manually contoured and labeled by an expert and reviewed by a neuroradiologist. FMRIB’s Linear Image Registration Tool (FLIRT), the affine registration tool from the FSL software package was used to align the atlas with the Chiari subjects’ imaging. Then, a more exact local alignment was performed using the FMRIB Nonlinear Image Registration Tool (FNIRT), which is also available with FSL. It is noteworthy that non-linear alignment or registration is performed when subject imaging and the atlas imaging differ to such an extent that images must undergo greater manipulation and deformation to properly align with each other. The mask was subsequently used to segment the fossae of the study subjects. During automatic posterior fossa parcellation, voxels of the mask were labeled as gray matter, white matter, or CSF.

The authors also employed the open-source atlas-based segmentation software FreeSurfer (http://surfer.nmr.mgh.harvard.edu). FreeSurfer uses anatomical spatial relationships as well as intensity distributions or ranges of anatomical structures for segmentation. Hindbrain volume was determined through the use of tissue intensity distributions and anatomical brain relationships that have already been defined. Hindbrain volume was generated by combining the volume of labeled brain stem structures with the volumes of the cerebellar gray and white matter.

FreeSurfer compared to FSL produced significantly larger hindbrain tissue volumes, as it did not exclude the volume of the herniated tonsils. Moreover, FreeSurfer could not determine the actual PFV, compelling the researchers to use the PFV values generated by FSL when finding the crowdedness index (i.e. tissue volume/PFV). The Dice coefficients of all patients for manual and automated segmentation of the posterior fossa were greater than 0.95. The Dice coefficients for the crowdedness indices determined via FSL and FreeSurfer of all patients exceeded 0.93[67].

Employing a multi-atlas label fusion approach, Huo et al. introduced the NLSS (Non-Local Spatial STAPLE; a combination of Spatial STAPLE and Non-Local STAPLE) algorithm for the segmentation of the posterior fossa and the entire intracranial space [56].

Semi-automated segmentation was conducted by first aligning CT images to MR images. A threshold was applied to the CT images, thereby producing a skull mask. The total ICV label was generated and propagated to the MR imaging. Topology-preserving Geometric Deformable Model (TGDM) was used to segment the total ICV [68]. By combining the segmentation with the total ICV label, a PFV label was created. The PFV label was subsequently manually edited, producing an atlas. 20 subjects’ MR and CT imaging was used to generate 20 semi-manual atlases. 15 atlases were chosen and aligned with the target imaging. Notably, label fusion is designed to resolve voxel-wise label conflicts among registered atlases by combining each atlas’ segmentation results into a single volume.

The study’s authors have described the advantage of NLSS in brain segmentation previously [67]. Out of the 20 original atlases, 19 were used. FSL and FreeSurfer were also used to find the total ICV. In the study, NLSS achieved more accurate total ICVs following multi-atlas-based segmentation in comparison to FSL and FreeSurfer. The coefficient of determination (R^2^) for NLSS compared to semi-automated segmentation was 0.970, whereas the coefficients for FSL and FreeSurfer were 0.855 and 0.910 respectively.

Nowinski et al. generated a 3D atlas with parcellated white matter tracts to enable real-time study of individual tracts and the neuroanatomy [57]. The method utilized a three-step approach. DTI-MPRAGE midsaggital images from a single subject are initially registered manually with rigid transformation. Imaging alignment was confirmed via affine transformation in FreeSurfer. The next step involved 3D tract generation from DTI using the DTI Studio software package. By selecting initially, a high fractional anisotropy, a measurement used to help define white matter tracts, threshold as well as a low angle, posterior fossa white matter tract volumes were generated. The fractional anisotropy and angle were adjusted to produce tracts with an appropriate number of intertract connections. These segmentations were compared to MPRAGE white matter and edited accordingly.

Sullivan et al. studied structural MRI images of 833 subjects from the National Consortium on Alcohol and NeuroDevelopment in Adolescence (NCANDA) cohort. Mega cisterna magna was the most common anomaly, comprising 26.5 percent of such cases [58]. Following their previously described protocol [69], T1-weighted 3D images were subjected to skull stripping by applying majority voting to the maps extracted by the Robust Brain Extraction (ROBEX) method and FSL brain extraction tool. ICV and volumes of substructures were identified using the SRI24 atlas-based analysis pipeline [70] and FreeSurfer. The PFV and the percent CSF by volume of the cisterna magna were found to aid in the identification of cases of mega cisterna magna, determining that clinical correlation is required for patients with percentages ≥ 3 SD.

Meier et al. created an automated segmentation pipeline for the analysis of white matter lesions in multiple sclerosis [59]. The authors introduce the concept of dual sensitivity to tackle non-uniform noise-to-contrast ratios in 3 T-MRI. First, bias field correction (N4ITK algorithm, available through Insight Toolkit of NIH), a preprocessing step used to remove intensity gradients generated by strong magnetic fields in MRI, was used to eliminate intensity heterogeneity. FLAIR and T2-weighted sequences were aligned with a T1-weighted reference series via the Insight Segmentation and Registration Toolkit (ITK) [71]. After stripping the skull via the brain extraction tool (BET) from the software package FSL, FreeSurfer was used for anatomical parcellation. During parcellation, brain regions were labeled and tissue classes including CSF, white matter, and gray matter were segmented. Intensity normalization was performed using a custom method; see reference by Meier and Guttmann for details [72]. Subsequent steps included custom methods that were used to develop tissue probability maps of white matter, gray matter, and CSF. An expectation–maximization algorithm was then used to segment infratentorial and supratentorial lesions using intensity thresholds that were unique to each respective region. Manual segmentation by a physician-scientist was concordant with the automated method, as demonstrated by an intraclass correlation coefficient of 0.95.

Artificial neural networks contain units or nodes serving as “neurons” that are connected to other nodes, forming a trilayer-nodal structure [73]. The input layer receives information from the user and the output layer returns its response to the input data. A neural network will have many nodes arranged in a series of hidden layers (https://wiki.pathmind.com/neural-network). The input and output layers are in between these layers. The nodes inside these hidden layers are connected to nodes in layers before and after these layers, forming connections or “weights” that function like synapses. A deep neural network simply denotes having multiple hidden layers. The higher the weight, the more influence the transmitting node will have on the receiving node. At each node, weight is combined with input from the data used, determining the degree to which the input data is deemed useful for the network’s task. The method by which the network “learns” involves an adjustment of this weight to minimize the difference between the output predictions and the ground truth. This process, known as back-propagation, is iteratively repeated until the network’s results are optimized.

Lin et al. developed a deep learning-based fully automated method to visualize neurovascular compression by reconstructing trigeminal nerve, brainstem, and cerebrovasculature utilizing MRA imaging [60]. Based on a 3D convolutional neural network, the method attempts to address the limitations of previously described semi-automated methods and employs a two-step approach. Coarse segmentations are carried out initially to produce confidence maps of the target structures (e. g. trigeminal nerve) using a modified CS^2^Net as the backbone [74] followed by a refinement step that further defines the boundaries of the target tissues. CS^2^Net performs segmentation of curvilinear structures, utilizing encoder, channel and spatial attention, and decoder modules. The researchers compared the proposed method with several previously described automated segmentation methods (e.g. 3D U-Net) to generate volumes for the various target tissues. The Dice similarity coefficient for the trigeminal nerve was determined to be 0.8024 for the proposed method as compared to 0.7213 using the 3D U-Net segmentation.

Liu et al. developed a deep learning automated segmentation approach for skull removal and intracranial volume measurement [61]. Their proposed method extends a method originally proposed by Huo et al. [75], which employs 3D spatially localized atlas network tiles (SLANT). This approach enables the estimation of total ICV and PFV in skull-stripped (ssSLANT) and non-skull-stripped (nssSLANT) brains. A transfer learning method estimates the total intracranial volume and PFV labels in T1-weighted MRI. Pre-training of U-Net tiles via BrainCOLOR atlases without total ICV and PFV labels (generated by multi-atlas segmentation) was followed by a refinement step employing limited BrainCOLOR atlases to train additional labels. The method can be used to segment the whole brain and estimate brain volume with or without the skull. Whole brain segmentation performance was similar for ssSLANT and nssSLANT as demonstrated by comparable mean Dice similarity coefficient values of 0.778 and 0.782, respectively. Regarding segmentation of the posterior fossa, ssSLANT and nssSLANT achieved superior values (0.977 and 0.975, respectively) compared to NLSS (0.968).

The literature search revealed several reports describing techniques designed for automated identification and differentiation of posterior fossa tumors. The methods generally employ features such as texture and intensity of MR images for tumor diagnoses. Ahmed et al.’s algorithm used texture features (e.g. multi-fractional Brownian motion) to achieve the best segmentation results for T1-, and FLAIR-MRI while the fusion of level-set shape with intensity features worked best for T2-MRI [62].

Schmidt et al. developed an algorithm for automated white-matter lesion segmentation in MS with good sensitivity (> 0.43). FLAIR intensity distributions were determined for the CSF, gray matter, and white matter tissue classes. Belief maps were created via these distributions as well as the spatial probability of being white matter. The gray matter belief map was used to create the initial seeds for segmentation. Neighboring voxels were incorporated into the lesions based on an initial intensity threshold, which was iteratively adjusted, while also tracking the Dice coefficient. The study found good agreement between automated and manual segmentation, with an overall Dice coefficient for posterior fossa lesions of 0.94 [63].

### Cavalieri estimator and other techniques

Table 4 summarizes numerous miscellaneous techniques that have been used to study changes in posterior fossa volumes. Several studies employed the Cavalieri principle, a point-counting method used to approximate the volume of a structure in diagnostic imaging [76–79]. The method relies upon generating a grid of points for each image slice and counting the number of points that cover the given region of interest. Volume can then be found by multiplying the number of points by the area of each point and by the distance between parallel planes of points. The method was used by Vurdem et al. to assess the PFVs of Chiari malformation Type I patients compared to those of age- and sex-matched controls [80]. A grid test system was implemented with a 0.8 cm distance between points. Volume was calculated by first determining the square of the product of the “scale unit” and interpoint distance divided by scale length. The number of points that overlapped with the region of interest was multiplied by 5, which was the section thickness in mms. Finally, the previous two values were multiplied by each other to find the volume. Chiari patients were found to have smaller PFVs and larger ratios of cerebellar volume to PFV.

Another study used the Cavalieri principle to examine the posterior fossae of children with Costello syndrome [81]. Crowding of the posterior fossa was observed in all 7 Costello syndrome patients, with a low ratio of PFV to posterior fossa brain volume. Five out of seven children had tonsillar herniation at the time of the initial evaluation.

Trigylidas et al. have also used the point-counting method to elucidate the relationship between PFV, intracranial volume, and Chiari malformation [82]. A grid was placed over each imaging slice using an online grid generator. The dimensions of the grid were selected according to the particular scale of the imaging. The ratio of PFV to intracranial volume was significantly smaller for Chiari patients compared to controls. Yet, no significant difference in crowding of the posterior fossa was found between asymptomatic and symptomatic Chiari patients.

Dogan et al. used fetal 3D ultrasound and Virtual Organ Computer-aided AnaLysis (VOCAL™) to analyze the PFVs of fetuses with DWM. The approach calculates volume by rotating a region of interest (ROI) around an axis and converging the various planes. The study found that PFVs were significantly larger in subjects with DWM as well as mega cisterna magna (MCM) compared to control subjects (83).

#### Advantages and disadvantages of the methods investigated

To advance our collective understanding of neurological disorders that afflict the posterior fossa, researchers have utilized multiple segmentation techniques that have evolved over the past decades (Table 1, 2, 3 and 4). Manual segmentation, when performed by a clinician with expert anatomical knowledge, has remained the gold standard. Yet, most researchers acknowledge the fact that such a method is time-consuming and labor-intensive [54]. Automated and semi-automated segmentation techniques, once developed, have the potential to be highly efficient. Unfortunately, to validate the results of these techniques, a comparison to the “ground truth” must be made. This “ground truth” is achieved through expert manual segmentation.

**Table 3:** Automated segmentation of the posterior fossa: summary of studies, Imaging modalities, segmentation software and techniques

Ref	Study Type	Patients/Controls	Imaging, Methods, Segmentation Techniques and Software	Time/ computational power details	Segmentation/Study Significance and Outcome	Operated and supervised by
Bagci et al. [3]	Automated PF volumetry; applications to CM-I	14/3	T1-weighted MRI of 9 CM-1 patients used to generate atlas. 3D-Slicer. FSL	3.0 T MRI, TR/TE/TIof1900/2.89/900 m, thickness, 1 mm	Good agreement between the proposed method and manual segmentation [small relative percentage difference in volumes; high mean Dice coefficient (0.968)]	Neuroradiologist
Huo et al. [56]	Simultaneous total ICV and PFV estimation using multi-atlas label fusion	-/20	CT; T1-weighted MRI. Multi-atlas (atlases generated by semi-manual segmentation). NLSS algorithm. FreeSurfer, FSL	–––-	Acceptable performance in estimating total ICV and PFV; NLSS mean dice coefficient for PFV (0.968)	––––
Nowinski et al. [57]	3D interactive and stereotactic human brain atlas of white matter tracts	-	DTI, MPRAGE MRI. DTI Studio software package 2.10 [^1^]	3.0 T MRI, TR/TE 4,031/93 ms, thickness, 3 mm	Developed a stereotactic tract atlas with parcellated white matter for real time exploration	Scientist
Sullivan et al. [58]	Brain anomalies in healthy adolescents in the NCANDA cohort; PF CSF volumes to detect mega cisterna magna	-/26	3D T1-weighted MRI. SRI24 atlas-based analysis pipeline, FreeSurfer. See reference [^1^] for detailed method	3.0 T MRI, IR-SPGR: TR = 5.904 ms, TI = 400 ms, TE = 1.932 ms, MPRAGE: TR = 1900 ms, TI = 900 ms, TE = 2.92 ms	22 of the 26 subjects’ merit further review based on percent of CSF in the posterior-inferior-middle aspect of the posterior fossa (incidental findings)	Neuroradiologist
Meier et al. [59]	Dual-Sensitivity multiple sclerosis lesion and CSF segmentation for multichannel 3 T brain MRI	29/15	FLAIR, T1- and T2-weighted MRI. 3 T morphometry (3TM) pipeline	3.0 T MRI, T1: TE/TR = 2.96/2,300 ms, T2: TE/TR = 300/2,500 ms, T2‐FLAIR (TE/TR = 389/5,000 ms	Manual segmentation was concordant with the proposed automated method. Intraclass correlation for white matter lesions = 0.95	–––––
Lin et al. [60]	Deep learning/auto-segmentation of trigeminal nerve and cerebrovasculature in MR angiography	-/50	MRA. 2-stage neural network. Modified CS2-Net	––––––	50 MRA volumes were used to evaluate multi-tissue segmentation with an average Dice similarity coefficient, Hausdorff distance, and average surface distance error of 0.8645, 0.2414, and 0.4296, respectively	–––––-
Liu et al.[61]	Automatic total ICV estimation and whole brain segmentation	-	T1-weighted MRI. 3D spatially localized atlas network tiles (SLANT)	––––––-	Segmented whole brain, estimated brain volume with or without skull. PFV segmentation mean surface distance of 0.554 and 0.542 for nssSLANT and ssSLANT, respectively. Dice similarity coefficient values were 0.975 (nssSLANT) and 0.977 (ssSLANT). Mean ASIM values were 0.992 (nssSLANT) and 0.993 (ssSLANT)	––––––-
Ahmed et al. [62]	Using image features (texture, intensity, etc.) to differentiate PF tumor from normal tissue	6/-	T1- and T2-weighted MRI, FLAIR. Expectation–maximization (EM) algorithm	1.5 T MRI, T1: TR = 168 ms, TE = 8 ms, T2: TR = 6430, TE = 114 ms, thickness = 5 mm	Texture features e.g.multi-fractional Brownian motion) yields best segmentation results for T1-, and FLAIR-MRI; fusion of level-set shape with intensity features works best for T2 MRI	Radiologist
Schmidt et al. [63]	Detection of FLAIR-hyperintense white-matter lesions in MS	53/18	3D T1-weighted gradient echo and 3D FLAIR MRI. SPM8/VBM8 (algorithm in MATLAB)	3.0 T MRI, T1: TR = 9 ms, TE = 4 ms, T2FLAIR: TR = 10000 ms, TE = 140 ms, thickness = 3–6 mm	Developed algorithm for automated lesion detection w/ good sensitivity ≥ 0.43. Good agreement seen between automated and manual segmentation; lesions volume, Dice coefficient 0.94	––––––––––––
Rijken et al. [64]	CM-I development in children with craniosynostosis syndromes	28, 85/34	3D-SPGR T1-weighted MRI. Multi-atlas-based segmentation with manual correction [see. Ref. ^1^]. ITK-SNAP	1.5 T MRI, T1: TR = 9.9 ms, TE = 3.1 ms, thickness = 2 mm	Cerebellar volume and PFV cannot predict CM-I susceptibility of craniosynostosis patients, but their ratio was significantly greater than for controls	––––––-
Alperin et al.[66]	PF morphology and CSF physiology in CM-I	36/37	3-D T1-Weighted MRI. Atlas-based segmentation [see ref.^1^]	1.5 T MRI, T1: TE/TR = 4.2/10.056 ms, 3 T MRI, T1: TE/TR = 3.91/2,300 ms	The multivariate analyses identified that maximal cord displacement, PFV/ICV, and PF crowdedness best differentiate CM-I patients from controls (sensitivity of 97.3% and specificity of 100%)	Neuroradiologist

**Table 4:** Cavalieri estimator/miscellaneous techniques

Ref	Study Type	Patients/Controls	Imaging, Methods, Segmentation Techniques and Software	Time/ computational power details	Segmentation/Study Significance and Outcome	Operated and supervised by
Vurdem et al. [80]	Volume analysis of PF, cerebellum, and herniated tonsils via stereological methods in CM-I	30/25	T1- and T2-weighted MRI. Cavalieri principle	1.5 T MRI, T1:TR/TE: 425/17.5 ms, T2: TR/TE:7450/102 ms thickness:1.5	Stereological methods can measure PFV and cerebellum volume. The PFV in CM-I patients was smaller than controls (P < 0.05)	Radiologist
Calandrelli et al. [81]	Costello syndrome: analysis of the PF in children with PF crowding	7/7	T2-weighted MRI. Cavalieri principle	1.5 T MRI	Demonstrated PF crowding in all 7 patients and tonsillar herniation in 5	Neuroradiologist
Trigylidas et al. [82]	PF dimension and volume estimates in pediatric patients with CM-I	32/20	T2-weighted MRI. Cavalieri principle	1.5 T MRI	Smaller ratio of PFV to ICV seen in CM-I patients; no difference in PF crowding in symptomatic vs. asymptomatic patients	Neurosurgeon
Dogan et al. (83)	Virtual Organ Computer-aided Analysis (VOCAL™) of PFV in fetuses	17/99	3D ultrasound. VOCAL™	––––––––	PFV was significantly larger in DWM and MCM groups in comparison to the control group	–––––––––
Calandrelli et al. (84)	Achondroplasia in children and possible overcrowding of the PF	13/13	3D FSPGR T1-weighted MRI. ITK-SNAP. Cavalieri principle	1.5 T MRI	PFV and PF brain volume increased in achondroplastic children vs. controls (p = < 0.05), ratio of PF brain volume to PFV normal	Neuroradiologist

The segmentation of the posterior fossa has classically been difficult due to its position between other brain regions, as well as the presence of poorly demarcated transition zones between these areas. One such difficult-to-distinguish boundary includes the junction of the cerebellum and cerebrum, which has complicated automated edge detection [43]. Intensity contrast is frequently required for accurate segmentation and the lack of a clear intensity change in this area increases algorithms’ reliance on user input.

The State of semi-automated segmentation: Semi-automated segmentation is not only faster than manual segmentation but also more reproducible [45]. By requiring user input, semi-automated segmentation controls the quality of a researcher’s contours. Nevertheless, it is apparent from our review of the literature that there does not exist one single semi-automated software, open source or otherwise, that has been widely applied to the posterior fossa. Whereas the vast majority of researchers used MRI, including T1- and T2-weighted scans, no universal technique currently exists. Researchers frequently create their segmentation software in such programming languages as MATLAB and Interactive Data Language. From automated edge detection and thresholding to region growing, semi-automated segmentation relies upon several common techniques across a wide spectrum of software. Furthermore, open-source semi-automated segmentation software is available, including ITK-SNAP, allowing researchers the opportunity to utilize segmentation without a potentially extensive mathematical or computer science background. However, the user interface of this software may be complicated and require knowledge of segmentation algorithms. Even so, such programs have provided researchers with largely accurate volumes over a wide range of studies.

#### Automated segmentation approaches

Fully automated segmentation is a modern technology that has already achieved highly accurate PFVs and/or crowdedness indices via open-source software such as FSL and FreeSurfer. However, these atlas-based methods require expert manual segmentation of regions of interest to operate successfully. Due to inter-subject anatomical heterogeneity, multi-atlas segmentation techniques such as NLSS have been used to enhance accuracy. However, multiple strategies are taxing computationally [56]. It has been shown that combining deep learning with multi-atlas-based segmentation can drastically reduce computational time and further advance the field of automated segmentation.

#### Benefits, detriments, and applications of segmentation techniques

Both thresholding and edge detection rely upon particular intensity criteria to properly segment a region of interest. In the case of edge detection, a boundary must be located where voxel intensity values drastically change. Thresholding is susceptible to noise due to the need for intensity homogeneity within the region of interest. Anisotropic diffusion may be used to eliminate noise and ease the segmentation process. Other techniques, such as region growth also ultimately rely upon intensity. The overall segmentation depends upon the intensity of the initial voxel or seed chosen. Moreover, only voxels within a certain threshold-dependent intensity range are incorporated into the region, with certain programs also only adding voxels that are sufficiently connected to a certain number of adjacent voxels. Of the various software described in this review, each has particular characteristics that may aid researchers and clinicians in the segmentation technique selection process.

Based on our study, automated methods of volumetric segmentation, such as atlas-based tools and neural networks, have been demonstrated to have priority over semi-automated and manual methods. Atlas-based segmentation software, including FSL, produced accurate results consistently. Researchers should select methods that are available, comprehensible, and accurate. Fortunately, there are now several options available. However, manual segmentation is highly dependent on the operator's expertise and would only be considered the gold standard if performed by an expert, otherwise creating a complete contour can be time-consuming. On the other hand, semi-automated segmentation is reliable in certain cases. Hence, the selection of the best option for users depends on various factors and facilities. When choosing a segmentation technique or software, the primary considerations for researchers and clinicians are cost, ease of use, imaging requirements, and time. Some software may require coding skills or may not be available for free. We highly recommend further research with more cases to investigate the accuracy of each method for simultaneous measurement exclusively.

#### Limitations of the study

Publication bias and language limitations may have limited the yield of our systematic review. However, we intended to review the clinically useful software available for segmentation and it is expected that researchers would report on useful technologies that advance their practice. While other software packages may exist, we assume that they have not been refined enough to prove to be useful as a generalizable tool for others. The papers identified did not always provide readers with complete details regarding the authors’ methods. Statistical metrics of accuracy and reliability were not provided by certain studies and varied from paper to paper, thereby limiting our comparison of technologies to each other or the accuracy of any given software. We have also chosen to include papers that do not strictly segment the posterior fossa, but instead segment abnormalities or structures within the region, to provide as complete of a review as possible.

## Conclusions

Segmentation of the posterior fossa continues to evolve into a more automated, accurate, and efficient process. The potential research possibilities grow ever more expansively as semi-automated and fully automated open-source software is validated. Atlas-based and neural network-based automated segmentation are extremely promising methods that produce accurate results. Future evolution of segmentation technologies will undoubtedly yield superior results, which may be applied to posterior fossa-related pathologies. Medical professionals will save time and effort due to these advances.

## Data Availability

None.
